# Burden of Common Childhood Diseases in Relation to Improved Water, Sanitation, and Hygiene (WASH) among Nigerian Children

**DOI:** 10.3390/ijerph15061241

**Published:** 2018-06-12

**Authors:** Zhifei He, Ghose Bishwajit, Dongsheng Zou, Sanni Yaya, Zhaohui Cheng, Yan Zhou

**Affiliations:** 1 School of Politics and Public Administration, Southwest University of Political Science & Law, Chongqing 401120, China; houis123@163.com (Z.H.); mrzds023@163.com (D.Z.); 2 School of International Development and Global Studies, University of Ottawa, Ottawa, ON K1N 6N5, Canada; brammaputram@gmail.com (G.B.); sanni.yaya@uOttawa.ca (S.Y.); 3 Health Information Center, Chongqing 401120, China; czhbtx@163.com

**Keywords:** diarrhea, fever, cough, Nigeria, infant health

## Abstract

Having access to improved water, sanitation, and hygiene (WASH) facilities constitute a key component of healthy living and quality of life. Prolonged exposure to insanitary living conditions can significantly enhance the burden of infectious diseases among children and affect nutritional status and growth. In this study we examined the prevalence of some common infectious diseases/disease symptoms of childhood among under-five children in Nigeria, and the association between the occurrence of these diseases with household’s access to WASH facilities. Types of diseases used as outcome variables included diarrheal, and acute respiratory infections (fever and cough). Access to WASH facilities were defined by WHO classification. The association between diarrhoea, fever and chronic cough with sanitation, and hygiene was analyzed by logistic regression techniques. Results showed that the prevalence of diarrhoea, fever and cough was respectively 10.5% (95% CI = 9.7–2.0), 13.4% (95% CI = 11.9–14.8), and 10.4% (95% CI = 9.2–11.5). In the regression analysis, children in the households that lacked all three types of facilities were found to have respectively 1.32 [AOR = 1.329, 95% CI = 1.046–1.947], 1.24 [AOR = 1.242, 95% CI = 1.050–1.468] and 1.43 [AOR = 1.432, 95% CI = 1.113–2.902] times higher odds of suffering from diarrhea, fever and cough. The study concludes that unimproved WASH conditions is an important contributor to ARIs and diarrheal morbidities among Nigerian children. In light of these findings, it is recommended that programs targeting to reduce childhood morbidity and mortality from common infectious diseases should leverage equitable provision of WASH interventions.

## 1. Introduction

As one of the fastest urbanizing country in the continent, Nigeria is experiencing significant challenges to provide access to improved Water, sanitation, and hygiene (WASH) to the population. Uncontrolled urbanization affects sanitation mainly through overcrowding of communities, constraints to quality housing, and reduced freshwater availability due to increasing consumption of water and water-intensive goods and pollution. The situation is exacerbated by poor environmental management and regulation which are failing to prevent the pollution of fresh water resources by accumulation of household and industrial waste water effluents [1]. Water crisis is looming large in the rural areas as well which is felt especially during the dry seasons, leaving as high as 70% of the households in serious water insecurity [2]. Nigeria’s sanitation and water poverty have raised concerns among many and has been reported by numerous national and international agencies. As of 2015, respectively 69% and 57% of the population in the urban and rural areas faced chronic water shortage [3] with about another 100 million living without access to adequate sanitation facilities [4]. In contrast, findings from a multi-country study by the joint monitoring program of WHO and UNICEF reported that 75% of the population had improved water and 59% that had improved sanitation [5]. Lack of access to WASH has important implications for population health especially among children who are more susceptible to infection and higher risk of illness due to their underdeveloped immune system [6]. Water and sanitation induced illnesses among under-5 children is considered as a serious public health concern in Nigeria [4,7].

The importance of WASH on infant nutrition and health status, and early childhood development cannot be overemphasized. WASH, referred to as improved quantity and quality of water, sanitation, and hygiene is widely acknowledged as the most cost-effective strategy to reduce the burden of infant morbidity and mortality by limiting the vectors of transmission through multiple food, water and environmental routes [8,9,10]. Inadequate access to clean water, basic sanitation and poor hygiene practices cause nearly 90% of all deaths from diarrhea in children [11]. Promoting access to safe excreta disposal, basic hygiene practices such as hand washing with soap, and provision of safe water supply are regarded as key strategies to limit the burden of infectious diseases among children including soil-transmitted health infections and diarrhea [6]. According to some estimates, globally an estimated 2.4 million deaths (4.2% of all deaths) could have been averted a year through optimum use of WASH [12]. Results from a WHO analysis maintained that promoting access to safe water and better environmental conditions could prevent about 94% of all diarrheal diseases [13]. The poor WASH infrastructure in resource-poor settings e.g., sub-Saharan Africa is reflected through the indiscriminately high burden of diarrhea and acute respiratory infections or ARI (common cold, cough, fever) [14]. In Nigeria, for instance, about 130,000 deaths among children are attributable to water-borne infections [15]. The burden of diarrhea attributable deaths among under-5 children have been reported to be 150,000 per year [16].

During past few decades there has been an escalating healthcare concern regarding the rise of non-communicable chronic diseases in the Nigeria, accompanied by reduced attention and budgetary capacity for the persistently high child-mortality and poor WASH conditions. Nigeria has been a signatory of the United Nations Declaration of the Right to Water and regulates its WASH activities through the Federal Ministry of Water Resources (FMWR). In recent years FMWR has been strengthening its policy efforts for scaling up the water and sanitation sector, and have launched several comprehensive projects in joint collaboration with UNICEF WASH [16] and USAID [17]. Some of these projects are working with ambitious targets of reaching the entire population and help meet the water and sanitation related Sustainable Development Goals or SDGs (Goal 6) in the country. However, it is notable that despite being a large country (largest in Africa), and having large scale programs on child health and WASH, there remains the lack of comprehensive population based studies on their association, and thus potentially limiting the scope for informed policy making and programmatic approaches. Therefore, to address this gap we conducted the present study using a nationally representative data drawn from the Nigeria Demographic and Health Survey. Although the survey was conducted few years back (in 2013), it is expected that the present study can provide important insights for the ongoing projects on WASH and child health related projects in Nigeria as well as for other countries in sub-Saharan Africa.

## 2. Materials and Methods

### 2.1. The Survey and Sampling Design

Nigeria Demographic and Health Survey (NDHS) 2013 was the fourth of the kind in Nigeria which was implemented by the National Population Commission with the financial and technical assistance by Inner City Fund (ICF) International provisioned through the USAID-funded MEASURE DHS program. DHS surveys are cross-sectional, nationally representative that collect information on a wide range of public health related topics such as anthropometric, demographic, socioeconomic, family planning and domestic violence to name a few. The survey covered men and women aged between 15–49 years and under-5 children residing in non-institutional settings. For sampling, a three-staged stratified cluster design was employed which was based on a list enumeration area from the 2006 Population Census of the Federal Republic of Nigeria. Enumeration areas are systematically selected units from the localities, which constitute the local government areas. Local government areas are subdivisions of each of the 36 administrative states (including the Federal Capital Territory called Abuja) and classified under six developmental zones in the country. Enumeration areas were used to form the survey clusters called primary sampling units. NDHS 2013 consisted of 904 clusters (372 in urban areas and 532 in rural areas) encompassing a total of 40,320 households from which 38,948 women were successfully interviewed with a response rate of 98%. Fieldwork lasted from 15 February 2013 to the end of May of the same year, and was carried out by 36 interviewing teams in each state plus one in the Federal Capital Territory of Abuja. A more detailed version of the survey was published elsewhere [18].

### 2.2. Variables

The outcome variables were prevalence of self-reported in commonly occurring Acute Respiratory Infections (ARIs) e.g., diarrhea, fever and cough. Mothers were asked whether or not the child suffered from these conditions during the past two weeks, and had the options to answer as- ‘Yes’ or ‘No’. Diarrhoea refers to the passage of three or more loose or liquid stools per day (or more frequent passage than is normal for the individual [19].

The explanatory variables of interest were access to improved (1) water; (2) sanitation and hygiene practices. We used WHO guidelines to classify the type of water and sanitation facilities as improved/unimproved (Table 1). The dataset did not contain any hygiene related variable. As a proxy indicator we used the information on disposal of child’s excreta which was also assessed by WHO guidelines.

A set of confounding variables were included in the analysis as well based on their relevance in light of previous studies such as Sex (Female, Male), Age in months (1–12, 13–24, 25–36, 37–48, 49–59), Birthweight are low birth-weight (LBW) and normal birth-weight (NBW), Stunting (No/Yes), Type of residency (Urban/Rural), Parents education (No education/Primary/Secondary/Higher), Household wealth status (Poor/Non-Poor) and presence of other infants in the household (≤2/3–4/>4) [4,16,17,18].

For the calculation household wealth status, instead of direct income the volume of durable goods (e.g., TV, radio and bicycle) possessed by the household as well as and housing quality (e.g., type of floor, wall, and roof) are taken into consideration. Each item is assigned a factor score generated through principal component analysis (PCA) which are then summed and standardized for the households. The scores thus obtained from a continuous scale and subsequently categorized into quintiles to rank the household as poorest/poorer/middle/richer/richest to richest [20]. For the present study, households in lowest two categories were merged and categorized as poor, and those from middle to richest were merged as non-poor.

### 2.3. Data Analysis

Before the analysis we checked the dataset for presence of outliers, and potential multi-collinearity. Following that, the dataset was converted to a plan file as complex samples by adjusting for the sampling strata, primary sampling unit and sampling weight. This is because DHS surveys employ cluster sampling methods for sample selection which needs to be taken into consideration for all analyses. As the initial analysis, the basic socio-demographic characteristics of participants were presented in terms of frequencies and percentages. Prevalence rate were shown as percentages with 95% CIs. Following descriptive analysis, Chi square bivariate tests were performed to check for the significant associations between disease status and the indicators of WASH along with the covariates. Variables that were found to be significantly associated in the Chi square tests (*p* < 0.25) were retained for final regression analysis. In the final step, binary logistic regression model was used to calculate the odds ratios of the associations between diarrhoea, fever and cough with WASH indicators. Precisely, individual disease status was modelled as a function of the three WASH indicators and to the binary logistic regression, while adjusting for various demographic and socioeconomic parameters which were found (based on literature review) empirically and theoretically pertinent to the outcome and exposure variables. Results of regression analysis were presented as odds ratios along with their 95% CIs as in indicator of significance as well as precision of the OR values. For all associations *p*-value of < 0.05 was considered statistically significant. All analyses were performed with SPSS version 24.

#### Ethical Approval

The protocol of DHS surveys was approved by the Ethics Committee of ORC Macro Inc., Calverton, MD, USA. The study was based on analysis of anonymized secondary data available in the public domain of DHS, therefore no additional approval was necessary. However, approval for the reuse of the data was obtained by authors from DHS.

## 3. Results

### 3.1. Description of the Sample

As shown in Table 2, the study included a total of 24,802 infants with a mean age of 28 months (±17.28). Most of the infants were aged 1 year of below (21.7%), normal birth-weight (96.8%), located in the north-west region (36%), of rural origin (64.5%). Literacy rate was higher among fathers (61.2%) compared with mothers (51.6%). Over half (54.8%) of the households were non-poor and about two-third had a maximum of two children (66.4%).

Regarding household characteristics, respectively 49% and 44.2% of the households were lacking access to sanitary toilet and pure clean water facilities, and 35.2% households did not practice proper methods for disposal of child’s excreta. Significant variations were observed in the rate of access to the three types of facilities across various individual (birthweight and stunting), geographical and household level factors (parents’ educational attainment, wealth status and presence of other infants in the household).

Table 3 indicates that fever was the most common of all three illness types with a prevalence of fever 13.4% (95% CI = 11.9–14.8) followed by diarrhoea 10.5% (95% CI = 9.7–12.0) and cough 10.4% (95% CI = 9.2–11.5). The prevalence rates of diarrhoea, fever and cough were higher among those who were normal birth-weight, stunted, of rural origin, born to parents with no formal education (except for fever), and from households with poor wealth status and had more than four infants (except for fever).

**Figure 1:**
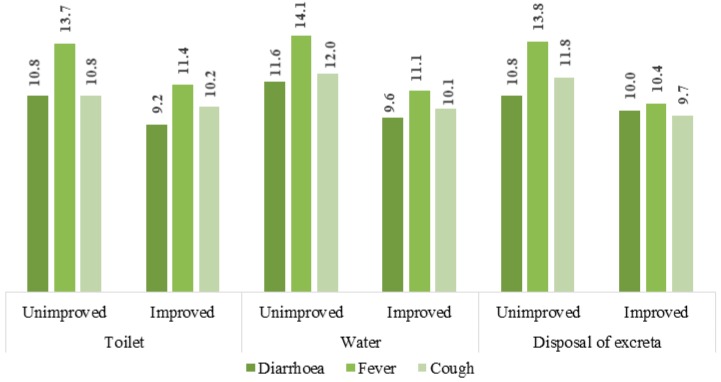
Prevalence of diarrhoea, fever and cough among under-5 children stratified by type of toilet, water, and child’s excreta disposal facilities.

Figure 1 illustrates the prevalence rates of diarrhoea, fever and chronic cough among households with and without access to improved sanitation, water and child excreta disposal facilities. It revealed that household that had improved facilities also had lower rates of prevalence of all three types of diseases.

**Figure 2:**
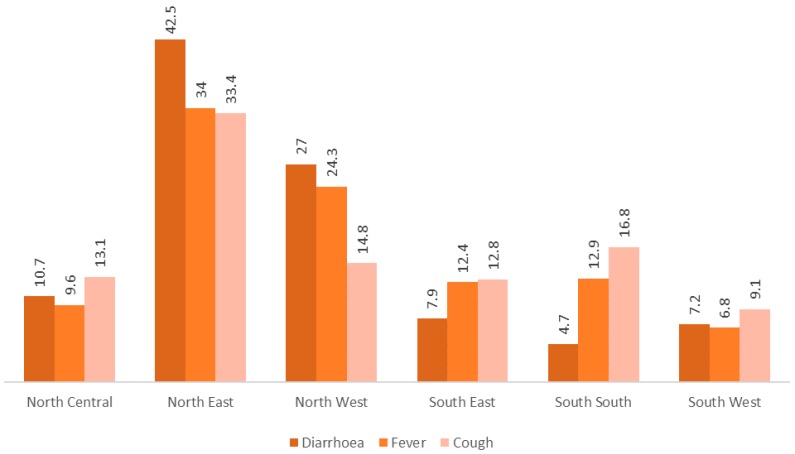
Prevalence of diarrhoea, fever and cough among under-5 children by region.

Figure 2 showed the regional disparities in the prevalence of diarrhoea, fever and cough. It reveals that the individual and combines prevalence of all these three diseases tended to be highest in the Northeast region and lowest in the South West.

**Figure 3:**
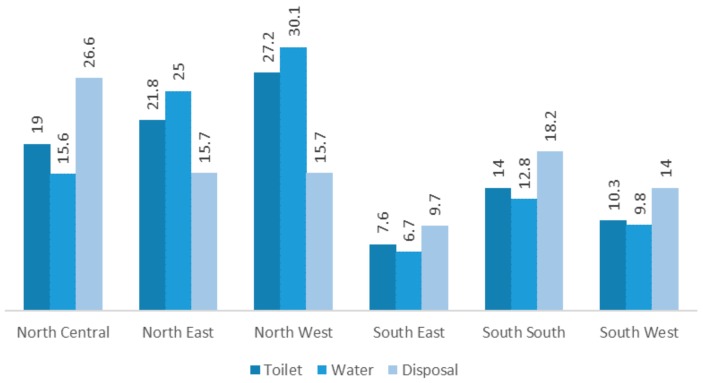
Percentage of households lacking access to improved toilet, water and child’s excreta disposal facilities by region.

Figure 3 illustrates the regional disparities in the accessibility to improved toilet, water and child’s excreta disposal facilities. It appears that the situation is worst in the North East and Northwest region, with South East and South West regions having comparatively better scenarios.

### 3.2. Regression Analysis

The results of multivariable regression measuring the associations between the three types of illnesses with household’s access to sanitation, clean water and child’s excreta disposal facilities were presented in Table 4. Access to sanitary toilet did not appear to be a significant predictor of any of the three diseases. The odds of suffering from diarrhoea were 1.6 [AOR = 1.602, 95% CI = 1.217–2.343, *p* < 0.001] and that of suffering from fever was 2.2 times [AOR = 2.193, 95% CI = 1.544–4.618, *p* < 0.001] as high among those with lack of access to clean water. Insanitary disposal of child’s excreta also showed 1.17 time [AOR = 1.172, 95% CI = 1.022–1.344, *p* < 0.001] higher odds of suffering from diarrhoea and 1.39 times [AOR = 1.393, 95% CI = 1.041–3.028] higher odds of suffering from fever. Finally, household that lacked all three types of facilities had respectively 1.33 [AOR = 1.329, 95% CI = 1.046–1.947, *p* < 0.001], 1.24 [AOR = 1.242, 95% CI = 1.050–1.468, *p* < 0.001] and 1.43 times [AOR = 1.432, 95% CI = 1.113–2.902, *p* < 0.001] higher odds of suffering from diarrhoea, fever and cough.

## 4. Discussion

In this study, attempts were made to provide an updated scenario of water and sanitation situation in Nigeria. Prevalence rates of three common childhood diseases namely diarrhea, fever and cough were also measured and was checked for their independent association with household’s access to improved water and sanitation facilities. Results indicate that fever was the most prevalent of the three diseases followed by diarrhoea and cough. In line with previous reports, the prevalence rates were higher among households that lacked access to improved WASH facilities [21]. In the bivariate analysis; stunting, parents’ educational achievement and household wealth status also appeared to be significantly associated especially with diarrhoea and cough. These observations are quite as expected because less educated parents are more likely to be unaware of the health risks associated with substandard water and sanitation quality and show poor hygiene behavior [22,23]. Higher socioeconomic status can act as a strong enabling factor for the utilization of WASH technologies, as well as a determinant of better self-efficacy for proper hygiene practices. Bivariate analysis also indicated a significant relationship between access to improved water and sanitation and the prevalence of the three types of diseases. The negative impact of poor WASH conditions on child growth and development have been shown to result from sustained exposure to enteric pathogens as well as various social and economic mechanisms [14,24].

Important regional variations were also noted in the prevalence of all three diseases, and of household’s access to improved WASH facilities. It appeared that household in the North East and Northwest regions share the highest burden of the diseases and have the lowest rate of access to WASH facilities. These findings are hard to explain in light of the current analysis, however can be implicated to the socioeconomic disparities across the six regions. There has been a marked North/South polarity in the country in terms of infrastructure, sociopolitical prosperity and human development indicators since the colonial times [25]. Hence, it is very likely that the relatively deeper impoverishment is the North partly accounts for the poorer health indicators. This finding therefore embodies an important message for the ongoing WASH projects in the country as conflicting geopolitical interests can significantly hamper the success of all development efforts.

The results also revealed statistically significant associations between the lack of WASH facilities with the occurrence of diarrhoea, fever and cough. These findings are similar with those from an Ethiopian study conducted among children (aged 0–50 months) in the slum areas of Addis Ababa. The study reported a diarrheal prevalence of 11.9% which is close to that in Nigeria. However, the rate of access to improved sanitation facilities was remarkably low (5.4%), perhaps because of study areas being slums, and was found to be a strong risk factor for diarrhoea [26]. In a nationally representative study in Uganda (Uganda Demographic and Health Survey 2011), a quarter of the infants were reported to have suffered from diarrhea during the past two weeks, a significant proportions of which were living in poorest WASH conditions [27,28]. Another Nigerian study showed evidences of infant mortality risks of from both unimproved water and sanitation [4]. Thus, the findings of the present study add further evidence to the current literature, and calls for heightened stress on WASH.

It is evident that as one of the fastest growing economy in the world, Nigeria is undergoing rapid demographic and epidemiological transitions in terms of the emergence of diseases and risk factors, changing trends in morbidity and mortality from certain disease groups. These altogether translate to mounting challenges for the healthcare sector due mainly to financial and infrastructure constraints to address the rising healthcare needs of the population. From this perspective, promoting WASH facilities and their proper utilization offer a key opportunity to minimize healthcare related constraints and promote population health in resource-poor settings [12]. This is particularly the case with respect to children’s health who are usually the most at-risk group for infectious disease and malnutrition related morbidities [29]. There are also documented evidences that poor WASH is associated with increased vulnerabilities to epidemics as widespread as HIV/AIDS [30] and tuberculosis [31]. It is therefore an urgent public health imperative to scale up WASH initiatives by ensuring multi-sectoral collaboration, especially by establishing closer cooperation between WASH and childcare stakeholders [6]. It is hoped that the current study provides important insights for researchers and interest groups involved in policy making in the areas of child health and sanitation issues. As a general recommendation for program managers and policy makers, special emphasis needs to be given on the underlying sociopolitical factors that engender inequality and barrier progress to the health promotion programs in the country since lack of organizational accountability and transparency is known to be rife among both public and private sectors [32].

As far as we are concerned, this is the first study to report on the relationship between WASH and diarrhea and selected ARIs. The data were cross-sectional, however large enough to help make meaningful conclusions, and more so since the samples were selected nationwide. One particular strength is that that analysis was adjusted for several important confounders including stunting and birth weight which have been found to be strong predictors of diarrhoea. Apart from that, we also considered three different types of communicable diseases instead of diarrhoea alone which can serve as a good reference for future researches in this area. Among the limitations were the self-reported nature of the variables that incurs the risks of recall and reporting biases, and lack of information on medication and presence of other disease conditions that could have influenced the strength of the association to certain degrees. Also, only a small of range of indicators were available for WASH as few others e.g., handwashing was not possible to include due to very limited amount of observations. As the data were secondary, we were unable to account for several important determinants of WASH such as gender equity, cultural and behavioural practices which need to explored through in-depth qualitative investigations. Further studies should be performed by considering a wider range of infectious diseases and using more proximate indicators of WASH to develop a deeper understanding of the causal relationships.

## 5. Conclusions

Based on secondary analysis of nationally representative data from Nigeria Demographic and Health Survey, the study drew several important conclusions which might of high interest for child health and WASH related stakeholders in the country. The magnitude of the burden of diarrhoea, fever and cough were alarming, as was the prevalence of households living without improved WASH facilities. Of particular concern was the presence of marked regional (North/South) disparities in WASH condition and morbidity of the diseases. Not having access to WASH facilities also appeared to be an important predictor of the disease conditions under study. In light of the current scenario, it is perceivable that special efforts will be required to ensure optimum WASH coverage for huge proportion of the population to produce observable benefits on child health outcomes. Qualitative studies are required to investigate the gender, cultural and behavioural aspects of WASH with respect to common infectious diseases among under-5 children.

## Figures and Tables

**Table 1:** WHO classification of improved sanitation and water supply.

	Unimproved	Improved
Sanitation + Child’s excreta disposal facilities	Unimproved sanitation facilities: do not ensure hygienic separation of human excreta from human contact. Unimproved facilities include pit latrines without a slab or platform, hanging latrines and bucket latrines.	Improved sanitation facilities: ensure hygienic separation of human excreta from human contact. They are use of the following facilities: Flush/pour flush to: piped sewer system, septic tank, pit latrine; Ventilated improved pit (VIP) latrine, Pit latrine with slab, Composting toilet.
Water	Unimproved drinking-water sources: Unprotected dug well, unprotected spring, cart with small tank/drum, surface water (river, dam, lake, pond, stream, canal, irrigation channels), and bottled water.	Other improved drinking-water sources: Public taps or standpipes, tube wells or boreholes, protected dug wells, protected springs or rainwater collection. Piped water on premises: Piped household water connection located inside the user’s dwelling, plot or yard.

Source: WHO/UNICEF Joint Monitoring Programme for Water Supply and Sanitation. ISBN 978 92 4 156395 6 (NLM classification: WA 670).

**Table 2:** Description of the sample children in NDHS 2013.

Variables	Total	Households Lacking Improved		
	(*N* = 24,802)	Toilet Facilities %	Water Facilities %	Child’s Excreta Disposal Facilities %
	*N* (%)	49.0	44.2	35.2
*Demographics*				
**Sex**				
Female	12,333 (49.9)	50.1	50.4	50.1
Male	12,469 (50.1)	49.9	49.6	49.9
		NS	NS	NS
**Age (28/17.28)**				
1–12 months	6131 (21.7)	24.7	25.1	25.0
13–24 months	5189 (20.5)	21.2	20.6	21.9
25–36 months	4616 (19.1)	18.6	18.7	17.7
37–48 months	4764 (19.6)	18.9	18.8	19.1
49–59 months	4102 (19.1)	16.7	16.8	16.3
		NS	NS	NS
**Birthweight**				
LBW	532 (3.2)	4.9	4.7	5.9
NBW	24,270 (96.8)	95.1	95.3	94.1
*p*		<0.0001	0.003	<0.0001
**Stunted**				
No	6909 (28.3)	31.7	31.3	25.3
Yes	17,893 (71.7)	68.3	68.7	74.7
*p*		<0.0001	<0.0001	<0.0001
*Geographic factors*				
**Type of place of residence**				
Urban	8342 (35.5)	17.9	20.6	25.8
Rural	16,460 (64.5)	82.1	79.4	74.2
*p*		<0.0001	<0.0001	<0.0001
*Household socioeconomic details*				
**Mother’s education**				
No education	11,365 (48.4)	56.4	58.5	43.8
Primary	5084 (19.3)	21.8	18.7	23.9
Secondary/Higher	8353 (32.3)	21.8	22.8	32.4
*p*		<0.0001	<0.0001	<0.0001
**Father’s education**				
No education	9142 (38.8)	45.9	47.4	36.3
Primary	4888 (19.1)	21.9	19.3	21.7
Secondary/Higher	10,772 (42.1)	32.2	33.3	42.0
*p*		<0.0001	<0.0001	<0.0001
**Wealth status**				
Poor	10,986 (45.2)	63.2	63.2	52.1
Non-Poor	13,816 (54.8)	36.8	36.8	47.9
*p*		<0.0001	<0.0001	0.063
**Infants in household**				
≤2	16,473 (66.4)	64.6	65.0	68.5
3–4	6981 (28.1)	29.1	28.7	27.2
>4	1348 (5.4)	6.3	6.3	4.3
*p*		0.014	0.039	0.001

N.B. NDHS = Nigeria Demographic and Health Survey, CI = Confidence interval.

**Table 3:** Weighted prevalence of self-reported Diarrhoea, Fever and Cough during last two-weeks among under-five children, NDHS 2013.

Variables	Diarrhoea	Fever	Cough
	10.5 (9.7–12.0)	13.4 (11.9–14.8)	10.4 (9.2–11.5)
**Sex**			
Female	49.8 (47.6–52.0)	48.7 (46.6–50.9)	49.7 (47.4–52.1)
Male	50.2 (48.0–52.4)	51.3 (49.1–53.4)	50.3 (47.9–52.6)
*p*	0.049	NS	Ns
**Age**			
1–12 months	23.4 (21.5–25.4)	25.0 (23.4–26.8)	24.5 (22.5–26.6)
13–24 months	21.7 (19.7–23.8)	20.1 (18.6–21.8)	21.7 (19.8–23.8)
25–36 months	18.2 (16.4–20.1)	19.2 (17.7–20.9)	19.2 (17.4–21.1)
37–48 months	21.6 (19.8–23.4)	20.4 (18.9–21.9)	20.2 (18.5–22.1)
49–59 months	15.2 (13.5–17.1)	15.2 (13.8–16.8)	14.4 (12.8–16.1)
	NS	NS	0.126
**Birthweight**			
LBW	2.8 (1.9–4.1)	0.9 (0.5–1.4)	7.2 (5.0–10.3)
NBW	97.2 (95.9–98.1)	99.1 (98.6–99.5)	92.8 (89.7–95.0)
	NS	<0.0001	<0.0001
**Stunted**			
No	34.8 (32.6–37.0)	30.8 (28.8–32.9)	25.7 (23.5–28.1)
Yes	65.2 (63.0–67.4)	69.2 (67.1–71.2)	74.3 (71.9–76.5)
*p*	<0.0001	0.0007	0.007
**Type of place of residence**			
Urban	31.7 (28.2–35.4)	33.7 (30.3–37.2)	40.5 (36.7–44.4)
Rural	68.3 (64.6–71.8)	66.3 (62.8–69.7)	59.5 (55.6–63.3)
*p*	0.013	<0.0001	0.003
**Mother’s education**			
No education	56.0 (52.5–59.4)	48.2 (45.0–51.5)	43.4 (40.2–46.8)
Primary	18.4 (16.4–20.6)	19.3 (17.4–21.5)	35.0 (31.8–38.3)
Secondary/Higher	25.6 (22.9–28.5)	32.4 (29.6–35.4)	21.6 (19.3–24.0)
*p*	<0.0001	NS	<0.0001
**Father’s education**			
No education	45.0 (41.3–48.8)	40.0 (36.5–43.5)	51.5 (48.3–54.7)
Primary	18.9 (16.8–21.1)	19.6 (17.7–21.7)	28.9 (25.7–32.2)
Secondary/Higher	36.1 (33.0–39.4)	40.4 (37.5–43.4)	19.6 (17.6–21.9)
*p*	<0.0001	NS	<0.0001
**Wealth status**			
Poor	53.9 (49.8–57.9)	51.7 (47.9–55.4)	60.8 (57.0–64.5)
Non-Poor	46.1 (42.1–50.2)	48.3 (44.6–52.1)	39.2 (35.5–43.0)
*p*	<0.0001	0.071	<0.0001
**Number of children 5 and under in household**			
≤2	5.8 (4.5–7.4)	4.8 (3.4–6.6)	3.4 (2.3–4.9)
3–4	30.8 (28.7–32.9)	27.6 (25.4–29.8)	25.9 (23.4–28.4)
>4	63.4 (60.8–66.0)	67.6 (65.0–70.2)	70.7 (67.9–73.4)
*p*	0.039	NS	<0.0001

NDHS = Nigeria Demographic and Health Survey. The 95%CI are presented between brackets.

**Table 4:** Regression analysis on the association between lack of access to toilet, water and child excreta disposal facilities with the three types of diseases.

Variables	Diarrhoea		Fever		Cough	
	COR	AOR	COR	AOR	COR	AOR
**Toilet facilities (yes)**						
No	1.104 (0.950–1.283)	0.972 (0.841–1.124)	1.054 (0.456–2.438)	1.029 (0.480–2.203)	1.240 (0.629–2.443)	1.149 (0.627–2.107)
**Water facilities (yes)**						
No	**1.179** **(1.010–1.376)**	**1.602** **(1.217–2.343)**	1.428 (0.616–3.311)	**2.193** **(1.544–4.618)**	1.310 (0.721–2.379)	1.059 (0.924–1.214)
**Child’s excreta** **Disposal facilities (yes)**						
No	0.927 (0.804–1.069)	**1.172** **(1.022–1.344)**	1.013 (0.442–2.325)	**1.393** **(1.041–3.028)**	1.388 (0.771–2.498)	1.245 (0.673–2.303)
**Toilet + water + disposal facilities (yes)**						
No	1.137 (0.968–1.336)	**1.329** **(1.046–1.947)**	1.180 (0.568–2.449)	**1.242** **(1.050–1.468)**	**1.220** **(1.050–1.418)**	**1.432** **(1.113–2.902)**

N.B. AOR/COR = Adjusted/Crude odds ratio. (yes) = Reference category. Bold numbers indicate significant associations (*p* < 0.05). The 95%CI are presented between brackets.

## Abbreviations

ARIs	Acute Respiratory Infections
DHS	Demographic and Health Survey
SDGs	Sustainable Development Goals
WASH	Water, Sanitation, and Hygiene
WHO	World Health Organization.
LBW	Low Birthweight
NBW	Normal Birthweight
